# A Novel Multi-Biomarker Assay for Non-Invasive Quantitative Monitoring of Kidney Injury

**DOI:** 10.3390/jcm8040499

**Published:** 2019-04-12

**Authors:** Drew Watson, Joshua Y. C. Yang, Reuben D. Sarwal, Tara K. Sigdel, Juliane M. Liberto, Izabella Damm, Victoria Louie, Shristi Sigdel, Devon Livingstone, Katherine Soh, Arjun Chakraborty, Michael Liang, Pei-Chen Lin, Minnie M. Sarwal

**Affiliations:** 1 KIT Bio, 665 3rd Street, San Francisco, CA 94107, USA; drew@kit.bio (D.W.); joshua.yang@ucsf.edu (J.Y.C.Y.); 2 Department of Surgery, University of California San Francisco, San Francisco, CA 94143, USA; reuben.sarwal@ucsf.edu (R.D.S.); tara.sigdel@ucsf.edu (T.K.S.); juliane.liberto@ucsf.edu (J.M.L.); izabella.damm@ucsfmedctr.org (I.D.); victorialouie11@gmail.com (V.L.); shristisigdel@gmail.com (S.S.); peichen@berkeley.edu (P.-C.L.); 3 Masters in Translational Medicine Program, University of California Berkeley, Berkeley, CA 94720, USA; devonlivingstone@berkeley.edu (D.L.); katherine_soh@berkeley.edu (K.S.); archakra@berkeley.edu (A.C.); liangmichael@berkeley.edu (M.L.)

**Keywords:** KIT Assay, chronic kidney disease, biomarker, non-invasive, urine, eGFR, cfDNA

## Abstract

The current standard of care measures for kidney function, proteinuria, and serum creatinine (SCr) are poor predictors of early-stage kidney disease. Measures that can detect chronic kidney disease in its earlier stages are needed to enable therapeutic intervention and reduce adverse outcomes of chronic kidney disease. We have developed the Kidney Injury Test (KIT) and a novel KIT Score based on the composite measurement and validation of multiple biomarkers across a unique set of 397 urine samples. The test is performed on urine samples that require no processing at the site of collection and without target sequencing or amplification. We sought to verify that the pre-defined KIT test, KIT Score, and clinical thresholds correlate with established chronic kidney disease (CKD) and may provide predictive information on early kidney injury status above and beyond proteinuria and renal function measurements alone. Statistical analyses across six DNA, protein, and metabolite markers were performed on a subset of residual spot urine samples with CKD that met assay performance quality controls from patients attending the clinical labs at the University of California, San Francisco (UCSF) as part of an ongoing IRB-approved prospective study. Inclusion criteria included selection of patients with confirmed CKD and normal healthy controls; exclusion criteria included incomplete or missing information for sample classification, logistical delays in transport/processing of urine samples or low sample volume, and acute kidney injury. Multivariate logistic regression of kidney injury status and likelihood ratio statistics were used to assess the contribution of the KIT Score for prediction of kidney injury status and stage of CKD as well as assess the potential contribution of the KIT Score for detection of early-stage CKD above and beyond traditional measures of renal function. Urine samples were processed by a proprietary immunoprobe for measuring cell-free DNA (cfDNA), methylated cfDNA, clusterin, CXCL10, total protein, and creatinine. The KIT Score and stratified KIT Score Risk Group (high versus low) had a sensitivity and specificity for detection of kidney injury status (healthy or CKD) of 97.3% (95% CI: 94.6–99.3%) and 94.1% (95% CI: 82.3–100%). In addition, in patients with normal renal function (estimated glomerular filtration rate (eGFR) ≥ 90), the KIT Score clearly identifies those with predisposing risk factors for CKD, which could not be detected by eGFR or proteinuria (*p* < 0.001). The KIT Score uncovers a burden of kidney injury that may yet be incompletely recognized, opening the door for earlier detection, intervention and preservation of renal function.

## 1. Introduction

Chronic kidney disease (CKD) is a worldwide public health problem, with a global burden of 850 million people. CKD is now being recognized as a “hidden epidemic”, as most patients with early stages of CKD (~11% of the world’s adult population) go unrecognized, as they do not have symptoms and are unaware of their increased risk for heart problems, infections, hospitalization and kidney failure. Hence, CKD is often not considered as a major health issue, even though it affects twice the number of diabetics (422 million) and over 20 times the number of people with cancer (42 million) or HIV/AIDS (36.7 million).

CKD is defined as reduced renal reserve, persisting for ≥3 months and/or a prior diagnosis of a condition with known risk for kidney damage [1]. CKD is defined in a five-stage classification system for the assessment of kidney injury as patients advance towards the need for dialysis or transplantation [2]. The current standard of care tests, the blood-based serum creatinine (SCr) assay (and its use to calculate the estimated glomerular filtration rate or eGFR [3]) and protein in the urine, detect CKD 3–5 (eGFR of ≤60 mL/min/1.73 m^2^ of body surface area). However, most cases of CKD 1–2 (CKD >60 mL/min/1.73 m^2^ of body surface area) go undetected. The eGFR has poor sensitivity for early CKD detection, as the inherent redundancy of renal reserve masks the early rise in SCr. Kidney biopsies, often triggered with more advanced stages of CKD, have value to assess pathology, but are invasive, expensive [4], and cannot be utilized for serial monitoring of CKD progression.

Multiple triggers and risk factors start the cascade of kidney damage in CKD, with progression to kidney failure (CKD 5). There are over 10 million people worldwide in need of kidney salvage therapy with lifelong support from dialysis or kidney transplantation. Though dialysis and transplant provide kidney functional replacement, they are both still encumbered by varying adverse impacts on patient morbidity and mortality. There is also marked impact of CKD on other organ systems in the body; CKD patients have higher risks of infections, cardiovascular deaths and cancer [5].

Early detection of kidney damage is crucial, as treatment in CKD 1–2 can result in renal preservation by either reversing or slowing the progression of kidney damage [6]. Currently available urine assays are unable to detect low levels of kidney injury [7,8,9,10,11,12,13,14] and have mainly been evaluated in acute kidney injury (AKI), with poor discrimination for CKD [9]. Thus, there is a critical unmet need to develop sensitive, quantitative, non-invasive diagnostics to detect CKD 1–3. Early detection and early treatment of CKD would mitigate the global disease burden and reduce the massive financial burden on national health care budgets [15,16,17,18,19,20,21].

The primary aim of this study was to perform an assessment of multiple novel and known urine biomarker measurements to conduct an inexpensive, portable assay, called the Kidney Injury Test (KIT) in consecutive patients presenting with CKD at a large tertiary health care system, and develop and independently validate a quantitative KIT Score to reflect the different CKD stages. The KIT assay performance was also directly compared to the current standard of care tests (eGFR and proteinuria). A secondary aim was to explore the accuracy of the KIT assay to detect and quantitate kidney injury in CKD 1–2, i.e., subjects with normal eGFR (>60) but with predisposing risk factors for CKD. This report describes the process of biomarker selection, algorithm development and independent validation of the KIT Score as a novel diagnostic for the assessment and quantification of both early- and late- stage kidney damage. 

## 2. Experimental Section

### 2.1. Patient Selection

Residual, random spot urine was collected from 1169 sequential patients from the clinical labs at the University of California, San Francisco (UCSF), over 3 months, from October 2016 to January 2017, as part of routine clinical testing at the Parnassus and Mission Bay campus clinical labs. Sample selection for a diagnosis of CKD was enriched by selecting urine samples obtained from the nephrology, diabetes, or cardiology clinics at UCSF. To facilitate statistical power, the study was enriched for patients at increased risk with kidney disease, using the following criteria: the patient had a confirmed diagnosis of CKD (ICD10 code N18 [22]), no diagnosis of CKD but a current diagnosis of diabetes, hypertension or autoimmune disease, no CKD but a positive family history of CKD, no CKD but aged over 60, or from an ethnic minority status. Patients with AKI were not included in this study, with the following rationale: (1) this first study on KIT is focused on the development and validation of the quantitative and functional metrics of the KIT Score in a chronic continuum of kidney disease progression. AKI has a different trajectory with acute onset and resolution; (2) AKI may have complete or incomplete resolution of injury, thus the KIT Score reduction after resolution of AKI will become clinically interpretable when the KIT Score ranges have been set in this paper for different functional stages of CKD; (3) AKI is one cause of CKD risk, but the majority of causes of CKD, such as diabetes and hypertension, do not start with AKI. A study of the KIT Score in AKI is planned as a follow-up study.

For purposes of KIT Score development, the relevant cause of CKD or the predisposing risk factor for CKD was captured from electronic health records (EHR), together with demographic variables and SCr, to allow calculation of the eGFR [23]. For each patient, the presence and cause of kidney disease was confirmed through a clinical review of medical and laboratory records. The most common causes of CKD in this cohort were: immune-mediated systemic diseases that can cause renal injury (such as lupus nephritis, rheumatoid arthritis, Sjogren’s syndrome), hypertension, diabetes (type 1 and 2), glomerular disease (these cases were biopsy confirmed with IgA nephropathy, membranoproliferative glomerulonephritis or focal segmental glomerulosclerosis) and obstructive uropathy (neurogenic bladder, posterior urethral valves, and hydronephrosis). In the CKD patient cohort, 74.8% of patients had 2 or more clinical diagnoses that would be relevant for CKD development and progression, while 13.3% of patients had diabetes and hypertension as dual kidney injury risk factors in the absence of a clinical diagnosis of CKD, reflective of the high prevalence of these comorbidities in the general population. The cause of CKD was not filtered, and all contributing causes of CKD were captured. 

To avoid degradation of the biomarkers in the KIT assay, urine samples were either processed within an hour of collection or stored at 4 °C and processed within 24 h. Samples were discarded or not included for analysis in this study if the sample volume was less than 2 mL, there were insufficient data to confirm patient CKD diagnosis, or there was >24 h delay in sample processing due to logistical reasons. After the above filtering process, we had a final selection of 343 unique urine samples from 343 patients. We also obtained additional urine samples from 54 healthy controls selected from volunteers who had good health, with normal SCr, no proteinuria, and no identifiable CKD risk factors. The study adhered to the Declarations of Helsinki and Istanbul and was approved by the institutional review board of UCSF (IRB 16-21108), and the requirement for informed consent was waived by the IRB.

### 2.2. KIT Assay Methods

#### 2.2.1. Sample Processing

Urine samples were collected in sterile containers (clean catch, mid-stream void), irrespective of micro- or macroscopic hematuria or proteinuria. Urine samples were centrifuged at 2000× *g* for 30 min at 4 °C. The supernatant was separated from the urine pellet containing cells and cell debris. The pH of the supernatant was adjusted to 7.0 using Tris–HCl and stored at −80 °C in the UCSF Biorepository until further analysis. 

#### 2.2.2. KIT Biomarkers

KIT inputs normalized measurements of 6 primary urine biomarkers. The first biomarker was cell-free DNA (cfDNA): as a measure of the total apoptotic burden of kidney injury [24], measured by a proprietary 5’ biotinylated oligonucleotide complementary chemiluminescent immunoprobe to specifically target cfDNA fragments in kidney injury [25]. This approach overcomes the limitations of time-consuming sample processing, costly PCR amplification [26], SNP detection ([27,28] or DNA sequencing methods [29], otherwise employed to measure cfDNA in blood [27,30]. The additional ELISA-measured markers include: methylated cfDNA (m-cfDNA) to refine the proportion of cfDNA that may be more relevant to renal parenchymal injury [31,32]; CXCL10, as a marker of inflammation [33,34,35,36,37]; clusterin, as a marker of renal tubular injury [38,39]; total protein, as a late marker of glomerular injury [40,41]; and creatinine, as a normalizing marker as it can be impacted by body mass, nutrition and/or hydration and utilized to avoid the need for a timed urine collection [42,43]. 

### 2.3. Statistical Analysis

#### 2.3.1. KIT Score Development

Random sampling was used to split the 397-patient cohort into a training (*n* = 233, with 37 healthy controls) set, stratified by kidney injury status. Random forest modeling was used to identify relationships between the different markers for the detection of CKD with high sensitivity. Predictive models were trained using statistical and machine learning methods for development of the KIT Score algorithm, across all six selected DNA and protein biomarkers and multi-dimensional partition of these assay measurements were based on identified clinical thresholds by incorporating the SCr and additional known risk variables for CKD such as race, gender and age. Finally, a simple linear model incorporating the resulting partition was developed into the KIT Score with an additional threshold for the assessment of low and high risk of CKD.

#### 2.3.2. KIT Score Validation

An independent validation subset of 164 patients, with 17 healthy controls, was subsequently used to prospectively validate the pre-specified KIT assay and KIT Score. Logistic regression was used to compare the (full) model with the current standard of care tests, urine protein alone and eGFR. A *p*-value < 0.01 for the corresponding likelihood ratio test was considered significant. Logistic regression analyses were performed to assess the accuracy of the pre-specified KIT Score threshold for the categorical (low and high) KIT Score Risk Groups. The sensitivity and specificity of the resulting quantitative and qualitative KIT Score, along with 95% confidence intervals, were calculated.

*T*-test and logistic regression was used to compare the pre-defined KIT Score and model patient status (individuals predisposed to CKD versus healthy subjects), to assess its ability to distinguish subjects with early stages of CKD (eGFR 60–90, CKD 2) and normal eGFR but with predisposing risk factors for developing kidney damage (> 90, CKD 1), from healthy volunteers (eGFR > 90) who have no known predisposing risk factors. A *p*-value < 0.01 was considered significant. 

## 3. Results

**Figure 1:**
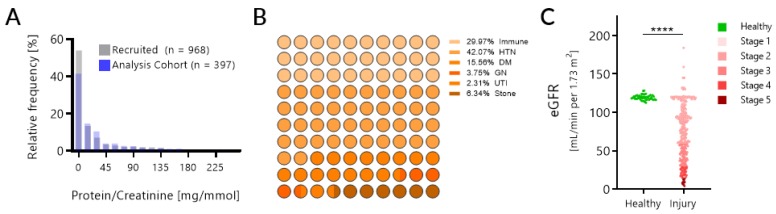
Cohort characteristics. (**A**) Proteinuria was assessed in the entire 968 patient samples and the distribution was plotted in grey. The distribution for the 397 patients selected for further biomarker analysis is overlaid in blue. (**B**) For the 343 patients out of the 397 with kidney injury, the contributing causes to CKD disease were plotted as a part of a whole plot, with the number indicating the proportion of patients with that etiology. Immune injuries include causes such as immunological glomerulonephritis, lupus, and rheumatoid arthritis (RA). Glomerulonephritic (GN) injuries include causes such as minimal change and IgA nephropathy. (**C**) The distribution of eGFR is depicted for the healthy and kidney injury subsets of 397 patients and color-coded by CKD stage.

**Figure 2:**
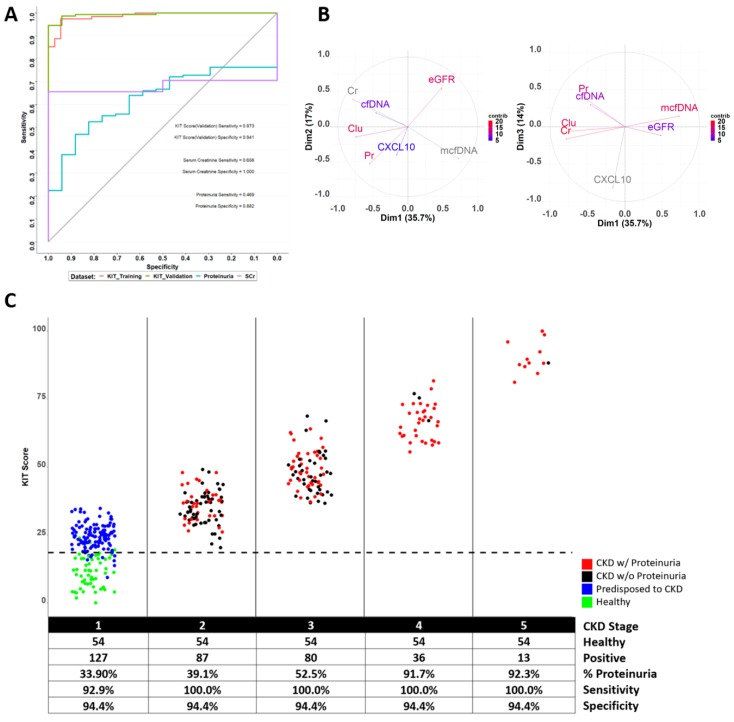
Cohort characteristics. Receiver operating characteristic curves and heat maps for kidney injury. (**A**) Receiver operating characteristic (ROC) curves for detection of kidney injury based on the KIT Score (training—orange and validation—green), serum creatinine (purple) and protein/creatine (aqua). (**B**) Principal component analysis loadings matrix for the KIT assay shows independent contributions of different biomarkers for the KIT Score and variances in biomarker data explained by each principle component. (**C**) KIT Score distribution as a function of CKD stage and proteinuria status (proteinuria positive, if urine protein μg/mL/urine creatine μg /mL ≥ 0.2). The CKD stages are shown in blocks on the X Axis: CKD 1 has no renal dysfunction and an eGFR of >90 mL/min/1.73 m^2^; CKD 2 corresponds to eGFR of 60–89 mL/min/1.73 m^2^; CKD 3 corresponds to moderate renal dysfunction, with an eGFR between 30 and 59 mL/min/1.73 m^2^. Patients with CKD 4 have severe renal dysfunction and an eGFR between 15 and 29 mL/min/1.73 m^2^. CKD 5, or end-stage renal disease (ESRD), corresponds to an eGFR <15 mL/min/1.73 m^2^ and generally leads to renal replacement therapy by dialysis or renal transplantation.

Of the 1169 patients recruited and urine samples collected, 201 samples met the exclusion criteria of low urine volume and were triaged from further analysis, leaving 968 patients (demographics in Table 1). The distribution of proteinuria in these 968 patients (0 to 5469.73; median (IQR) of 4.69 (0–39.11) mg/mmol) and the range of eGFR (calculated for all CKD patients [23] (Figure 1C)) highlights that we captured the spectrum of CKD disease (Figure 1A). From this cohort, 397 unique patients and samples survived the exclusion criteria (see Methods). Etiologies of their kidney injury encompassed a broad and multiple range of diseases, with hypertension as a contributing cause for 42% of patients (Figure 1B). Over 60% of patients had more than one contributing cause to their kidney injury. Given that this is a reality for most CKD patients, particularly as they progress to later stages of CKD, the KIT Score was modelled to detect kidney injury irrespective of the underlying cause. Despite proteinuria being the current gold standard for the non-invasive assessment of kidney injury, there was no correlation between the eGFR and the urinary protein/creatinine ratio (*R*^2^ = 0.0087) and, as shown by other investigators [44], proteinuria was poor at categorizing stages of CKD (Figure 2). 

The primary aim of this study was to develop a composite KIT Score scaling from 0 (low risk) to 100 (high risk) and prospectively assess the capability of a quantitative KIT Score for the detection of kidney injury with a high degree of sensitivity and specificity. We show that, though the current measures of renal function (eGFR and proteinuria) are predictive of *late*-stage CKD (Table 2), the quantitative KIT Score scaling from 0 (lowest risk) to 100 (highest risk) provides predictive information on kidney injury status above and beyond proteinuria and renal function alone (likelihood ratio $\chi_{1}^{2}$ = 52.507, *p*-value < 0.0001), and outperforms the current standard of care tests (Table 3). Urine creatinine is used for normalization purposes in the KIT algorithm, and controls for diurnal and hydration variations (thus obviating the need for any timed urine sampling) and was not significant for CKD stage in multivariate analyses (*p*-value = 0.2506).

We also developed a qualitative KIT Score, an approach that can have important implications for population kidney risk assessment. A pre-defined risk threshold of 18.5 was established in the training data, allowing the separation of the KIT Scores into low (≤18.5) and high risk (>18.5) groups of kidney injury. This was confirmed in the test-set to provide predictive information on kidney injury status above and beyond proteinuria and eGFR (likelihood ratio was $\chi_{1}^{2}$ = 44.244, *p*-value < 0.0001). 

The estimated sensitivity and specificity of the KIT Score for detection of CKD is 97.3% (bootstrap 95% CI: 94.6, 99.3%) and 94.1% (bootstrap 95% CI: 82.3, 100%), respectively. In contrast, the sensitivity and specificity of proteinuria was 46.9% (bootstrap 95% CI: 38.8, 55.8%) and 88.2% (bootstrap 95% CI: 70.6, 100%), respectively, for the same samples. Similarly, the sensitivity of SCr was 65.6% (bootstrap 95% CI: 57.4, 73.8%). The estimation of negative and positive predictive values of the KIT Score is dependent on the prevalence rate of the disease, thus the prevalence rate of hypertension can be used as an example. The prevalence of hypertension in the general US population is approximately 33%. Consequently, the estimated positive and negative predictive value of the quantitative KIT Score for hypertension would be ~89.1% and 98.2%, respectively.

We show the contribution of individual biomarkers to the KIT Score below (Table 4). The abundance of cfDNA in urine was highly variable across the different categories of renal disease and did not independently correlate with the stage of CKD. The correlations among the individual biomarkers were small (largest correlation *R*^2^ = 0.14 was between eGFR and protein), suggesting that each of the biomarkers is providing independent information towards the prediction of kidney injury status. Principal component analyses (PCA) (Figure 2B) elucidate the relationship of independent linear combinations of the biomarkers to the total variability in assay measurements and highlight the important contribution of the m-cfDNA, cf-DNA and CXCL10 as providing independent value in the generation of the composite KIT Score. Clusterin was the only kidney injury status biomarker that was not significant in multivariate analyses (*p*-value = 0.1671). These factors highlight that, despite published data on correlations between the abundance of blood cfDNA and kidney dysfunction [27,28], urinary cfDNA in these settings would not suffice alone, and there is value in including multiple biomarkers for developing the composite KIT score for CKD assessment.

CXCL10 is a key inflammatory cytokine dysregulated in immune-mediated renal injury [45,46,47,48,49,50,51]. To evaluate where CXCL10 identifies a specific cause of CKD, such as immune-mediated causes of renal injury from systemic lupus erythematosus, rheumatoid arthritis, antineutrophil cytoplasmic antibody (ANCA)-positive vasculitis, we evaluated which CKD categories had high abundance of urinary CXCL10. Interestingly, only ~30% of the immune-mediated cohort had very high CXCL10 values (>100 pg/mL), whereas, in many immune-mediated renal injury samples, these values were low and may reflect disease remission or immunotherapy exposure. To our surprise, ~30% of the hypertensive and diabetic cohorts also had high urinary CXCL10 levels, without any identified systemic immune-mediated diseases. There was no association of urinary CXCL10 levels and CKD stage.

Figure 2C displays the KIT Score, shown here as a function of CKD stage (based on the eGFR) and matched with presence (red dots) or absence (black dots) of proteinuria (threshold cutoff of urine protein/creatinine ratio of >0.2). The healthy controls are marked in green and are shown in a pre-CKD 1 or CKD 0 stage where the eGFR is >90 mL/min/1.73 m^2^ like in CKD 1, without proteinuria or identified risk for kidney injury. It is important to note that ~15% of the healthy controls tested here have a mild elevation in their KIT Score (just above the high-risk threshold of 18.5). At this stage of the study, we cannot identify if these healthy controls with higher KIT Scores have undetected risk factors for kidney injury, as all subjects were de-identified. 

Table 5 shows a breakdown of the mean KIT Score by CKD stage and for healthy volunteers, and we note a significant trajectory of increasing KIT Score by advancing CKD stage. The KIT Score can readily distinguish CKD 1 and 2 (28.9; 95% CI: 27.9, 29.9) from healthy volunteers without CKD (11.0; 95% CI: 9.5, 12.6); *p*-value < 0.00001). When compared to healthy controls, there is a strong association between the KIT Score and predisposition to kidney injury, even in subjects with normal renal function in CKD 1 (eGFR > 90), with a likelihood ratio of $\chi_{1}^{2}=148.4$ (LR *p*-value < 0.0001) and an assay threshold of 18.5 had a sensitivity and specificity of 92.9 (exact 95% CI: 87.0%, 96.7%) and 94.4% (exact 95% CI: 84.6, 98.8%), respectively. This same effect was further amplified for the KIT assays ability to detect both CKD 1 and 2, with a likelihood ratio $\chi_{1}^{2}=196.5$ (LR *p*-value < 0.0001), with the >18.5 KIT Score threshold having a sensitivity of 95.8% (exact 95% CI: 92.2, 98.1%) for early CKD detection. A total of 60% of patients in CKD 1 have elevated KIT Scores >18.5, even though they have “normal” renal function, and no proteinuria. These are exactly the target patients we want to capture with the KIT assay as their early-stage CKD would be undetected by the current standard of care tests. In fact, the KIT Score identifies 92% of patients in CKD 1 who have no proteinuria as having early kidney injury. In CKD 2–3, though approximately 60% of patients have no proteinuria, the KIT Score detects all patients as high risk and provides different quantitative measures for each patient’s CKD injury burden. In CKD 4–5, proteinuria and the KIT Scores are more concordant as kidney damage is advanced and proteinuria is recognized as a late marker of renal injury. 

Thus, these collective results clearly demonstrate that the assay achieves its primary and secondary objectives of predicting kidney injury with greater sensitivity than the current standard of care tests (eGFR, SCr, proteinuria), and, unlike our current clinical assays, the KIT Score can accurately identify very early stages of CKD in population screens.

## 4. Discussion

The driving force behind the development of the KIT assay and KIT Score was a recognition of the massive, and increasing, burden of unrecognized kidney injury [52], compounded by the weakness of the current standard of care tests [53] in uncovering the real and “hidden” burden of kidney disease. Tracking kidney damage in individuals by urine sampling has always been a highly desirable goal, as the biological signal from urine can provide a highly informative window into the health of the kidney. Nevertheless, a urine test to predict kidney injury, irrespective of underlying cause, remains a clinical unmet need and a biochemistry challenge to execute, as there are many interfering substances in urine such as proteases [54,55], resulting in biomarker degradation and high inter-individual variance in urine pH—all of which can confound the development of a robust laboratory assay. Targeted proteomic studies [56,57,58,59], fueled by protocolized urine sampling from prospective clinical trials, spanning a decade (in our laboratories at Stanford University and UCSF), has led to the development of standard operating procedures (SOPs) for urine collection, stabilization, processing, evaluation of interfering substances, preservation and transport from distant sites to a central processing lab [60]. In addition, over a decade of transcriptional and LC–MS/MS-based urine proteomic studies [48,50,58,61,62,63,64] have resulted in a deeper biological understanding of kidney injury across different causes of CKD, which drove the selection of biomarkers for inclusion in the KIT assay to represent injury across different intra-renal compartments.

This comprehensive search led us to six biomarkers: cfDNA, methylated cfDNA, clusterin, CXCL10, creatinine, and urinary protein. Cell-free DNA has been recognized as a sensitive marker of disease burden in the plasma of patients with autoimmune diseases [65,66] and tumors [67]. However, their utility in the plasma is limited in the setting of multiple diseases and morbidities, as total cfDNA burden would reflect the cumulative processes of various diseases, while organ or site-specific cell-free DNA measurement in the plasma requires advanced sequencing technologies and bioinformatics [68,69]. However, because cfDNA in the urine specifically reflects contributions from the kidney, the KIT cfDNA assay enables extremely sensitive detection of kidney injury via an inexpensive ELISA-based assay. The measurement of methylated fragments of cfDNA provides additional specificity regarding the type of injury. While global hypermethylation has been associated with immune-related kidney injury and increased fibrosis, global hypomethylation is associated with aging-related renal decline and renal ischemia-reperfusion injury [31,32,70]. As with our rationale in the measurement of cfDNA, we find that global changes in the methylation state of the cfDNA enable accurate discrimination between kidney disease states without the need for loci-specific sequencing or PCR.

CXCL10 has been well established as a marker of immune-mediated injury in a variety of contexts due to its role as a ligand for the CXCR3 receptor [33,34,37,45,51,71]. We previously showed that CXCL10 and cfDNA, as measured via the KIT assay, can detect chronic lung allograft dysfunction in lung transplantation as well as rejection in kidney transplantation [35,72,73]. Strikingly, what we found in the present study is that there is a significant number of patients with traditionally non-immune kidney diseases, such as hypertension and type 2 diabetes, that had elevated CXCL10, potentially indicating a broader utility in the detection of early-stage kidney injury. Prior studies have identified type 2 diabetes to have a significant CXCL10-mediated component [74] and have identified endothelial cell-produced CXCL10 as a contributor to essential hypertension [75]. Our findings suggest that CXCL10 can identify not only patients with immune-mediated kidney injury, but also those of other causes, ones that tend to present insidiously with late clinical symptoms relative to disease progression.

Urine total protein and clusterin are well-established markers of kidney dysfunction [38,40,41] and creatinine is well-validated in the field as a normalizing biomarker [42,43]. Surprisingly, we find that clusterin, after multivariate analyses, is not significant in our model of kidney injury in the context of our other biomarkers. This may be due to a number of causes, including its correlation to the total protein to creatinine ratio, the components of which are already included in the model [38], as well as the high spot variation due to the ultradian rhythms of the tightly correlated plasma and urinary levels of clusterin [76,77].

Early detection of kidney injury has very important ramifications on limiting the rate of CKD progression and positively impacting health care worldwide. As renal hemodynamic changes are acutely sensitive to systemic perturbations, improved management of the main underlying causes of CKD would be an immediate benefit, especially the improved and tight control of blood pressure [78], stability and tight control of hyperglycemia [79], and early detection and prompt immunomodulation for abrogation of renal inflammation and injury in immune-mediated systemic diseases such as systemic lupus erythematosus [80] and rheumatoid arthritis [81]. Persistent and early elevations of the score could trigger renal imaging to evaluate for obstructive uropathy, with prompt intervention to prevent high intra-renal pressure and progressive renal interstitial fibrosis and tubular drop out. Early detection of renal injury is most likely going to occur in the primary care setting, where the availability of a rapid throughput, simple assay with a quantitative kidney risk score read-out can trigger earlier referral to a nephrologist for blood pressure control, dietary modification, treatment of coronary and/or peripheral vascular disease, the underlying cause of the CKD and the consideration of renal preserving therapies [82,83]. The ability to quantitatively track the resolution of the KIT Score over time provides an opportunity to track kidney injury resolution. In addition to choices to use medications or support to better control the systemic disease, new reno-protective drugs, such as the SGLT2 inhibitors and others [84], further highlight how crucial it is to be able to detect very early kidney injury, treat and reverse it.

Delays in kidney injury detection and consequently patient referral is a significant obstacle to getting new patients into effective treatment regimens while they still have a chance to maximize the benefits of renal preserving therapies. To emphasize how many patients “miss” the opportunity for diagnosis of CKD in earlier stages of their disease, patients with ESRD use the emergency department at a rate six times higher than the national mean rate for US adults—half of which result in hospital admission [85]. Thus, in addition to using the KIT Score to track renal recovery, there is tremendous value to also using this assay to track renal injury progression so that interventions can be brought in as needed to stabilize KIT Score trajectory progression. The observation that a third of patients with CKD aetiology due to hypertension or diabetes also have a strong immune/inflammatory milieu as part of their renal injury suggests that appreciation of the biological heterogeneity of different categorical diseases by the individual biomarker values in the KIT assay, and other interrogative studies, will allow for more customized approaches to treatment for CKD patients. 

Ongoing studies are planned and underway whereby serial assessment of the pre-defined KIT Score with the pre-defined KIT assay will allow us to assess the value of individual biomarkers in the assay during renal injury and renal recovery. In addition, we are setting up collaborations with pharmaceutical partners where serial assessment of the KIT assay can be used as a means to non-invasively and accurately track for early kidney injury from drug nephrotoxicity, inherent to many immune-modulators, such as calcineurin inhibitors [86] and anti-TNFα agents [87]; chemotherapeutic agents such as cisplatin [88], aminoglycosides [89], and newer immunotherapies [90], as well as exposure to radionuclide contrast media for imaging purposes [91]. Additional cohorts are being assembled for analysis where patients have been followed up longitudinally over the course of CKD progression, which will allow us to better understand the granular trajectory of the KIT Score and possibly help refine CKD stages.

As the assay biomarkers were chosen and the assay was developed with the specific intent to detect very early kidney injury, we also observed that the KIT assay can detect that a small percentage of “normal” controls display urine KIT Scores that hover at the high-risk threshold of 18.5. Though not confirmed, it is possible that this is not assay noise and these cases are true positives with very early identification of kidney injury risk in the pre-CKD 1 or CKD 0 stage. As 96% of people with CKD do not realize they have it [92], one significant challenge in developing kidney injury models is the high likelihood that patients recruited as healthy controls actually have early-stage kidney disease. For example, the human longevity project, led by Craig Venter with the Health Nucleus test, extensively sequenced and performed additional screening tests of symptom-free adults and found clinical correlates of potential disease in 21% of their “healthy” study participants inclusive of urologic/renal diseases, suggesting the need for increased screening. This is especially true of diseases where early intervention can delay or even reverse disease progression. We find CKD and kidney injury as a whole to be an exemplary disease in which this is true, as numerous lifestyle and therapeutic interventions can prevent further progression of kidney function decline [6,82,83,92].

A limitation of the current study design is the use of a population enriched for CKD subjects from a tertiary care site. Subjects obtained from more screening and community settings may be more representative of the general population, particularly for the detection of early-stage CKD. Additionally, the study was cross-sectional and may not have fully represented early- and late-stage CKD. Longitudinal studies monitoring patients with signs of early-stage CKD are planned for the improved assessment of the early detection capabilities of the KIT Score and for the impact of early detection on CKD progression. Finally, it may be possible to further augment the KIT Score with additional biomarkers to allow improved differential diagnosis of CKD and minimize the need for serum creatinine and proteinuria measurement.

The positive economic impact of early kidney injury detection and its treatment cannot be underscored. Almost a third of the Medicare budget is devoted to the management of kidney injury and disease in the US. The loss of kidney function in CKD 5 adds an additional fiscal burden of ~$80,000 per year due to dialysis support. Although renal transplantation enables patients to come off dialysis, the shortage of renal donors, both living and cadaveric, renal transplantation, with an initial procedure cost of ~$100,000, followed by maintenance medication costs of ~$20,000/year, results in only a small dent in the dialysis Medicare budget [93]. The trajectory for the numbers of patients with CKD is expected to continue to rise worldwide, with greater trends in obesity, resulting in rising numbers of people with hypertension and diabetes [94]. In addition, ethnic variations in renal diseases drive national health care problems, requiring population screening with kidney biopsies for early detection of renal injury from IgA kidney disease in South-East Asia [95], where IgA kidney disease is the prime cause of renal failure. The inclusion of a sensitive non-invasive assay for renal injury, to replace invasive, high-cost, high-morbidity biopsy procedures, would result in major socio-economic benefits for these at-risk populations.

In conclusion, this study provides the blueprint for the KIT assay biomarkers, the KIT assay algorithm development process, the KIT Score definition and its performance for the early detection of kidney injury, and its direct comparison with the current standard of care tests. This study proposes that the KIT test could be used (1) as a screening test when there is a concern of co-morbidities that are known to increase the risk of CKD, such as cardiovascular morbidity, obesity, diabetes, without documentation of abnormal kidney function by current assays; (2) as an adjunctive test to monitor the burden and accurate progression of CKD in patients where the current standard of care tests have already detected renal injury, i.e., patients with known pre-existing CKD at different stages of kidney disease; (3) to allow for the sensitive assessment of recovery from kidney injury when exposed to different reno-protective therapies and management options. Further studies are needed, and planned, for the longitudinal screening of patients over time to better understand the natural history of the progression of CKD, the benefits of early detection, intervention and monitoring, and the infection points where CKD injury becomes fixed and progressive. 

## Figures and Tables

**Table 1:** Demographics and presenting features of the study cohort ^1^.

Variable	Sampled Cohort (*n* = 397) Median (Range)	Training Cohort (*n* = 233) Median (Range)	Test Cohort (*n* = 164) Median (Range)
**Age**, years	53 (2–98)	53 (2–94)	52 (4–98)
**Gender**, women, %	49%	47%	53%
**Race**, % AA	13.2%	12.9%	13.7%
**Proteinuria**, (mg/mmol creatinine)	74.56 (0–8239)	76.58 (0–8239)	62.12 (0–3135)
**Serum creatinine**, (mg/dL)	0.98 (0.31–9.36)	1.06 (0.31–9.36)	0.83 (0.32–7.13)
**Estimated glomerular filtration rate (eGFR)** (mL/min/1.73 m^2^)	85 (4–184)	73 (4–159)	94 (6–184)
**Cause of kidney injury**, %			
• Immunological	30.0%	34.9%	22.5%
• Hypertension	42.1%	42.6%	41.3%
• Diabetes	15.6%	14.8%	16.7%
• Glomerulonephritis (GN)	3.7%	1.0%	8.0%
• Urinary Tract Infection (UTI)	2.3%	1.9%	2.9%
• Kidney Stone	6.3%	4.8%	8.7%
**Stage of chronic kidney disease (CKD)**, %			
• Healthy	15.6%	15.3%	15.9%
• Stage 1	36.6%	28.2%	49.3%
• Stage 2	25.1%	26.8%	22.5%
• Stage 3	23.1%	26.3%	18.1%
• Stage 4	10.4%	12.4%	7.2%
• Stage 5	3.7%	5.3%	1.4%

^1^ No significant differences were identified between the training and test cohorts in the final selection of 397 unique patients, selected based on clear phenotypes of healthy control (*n* = 54) and overt injury (*n* = 343).

**Table 2:** Multivariate logistic regression of kidney injury status as assessed by SCr, eGFR and proteinuria.

Parameter	Estimate	df	s.e.	χ^2^	*p*-Value	OR	2.5%	97.5%
Intercept	44.6953	1	12.8490	12.0999	0.0005			
eGFR	−8.9508	1	2.6815	11.1422	0.0008	1.30 × 10^−4^	1.70 × 10^−7^	9.65 × 10^−3^
Proteinuria	0.4971	1	0.2787	3.1815	0.0745	1.64	1.01	3.03

Model likelihood ratio $\chi_{2}^{2}$ = 40.077, *p*-value < 0.0001. OR is odds ratio for increased risk of kidney injury per unit change in Ln(eGFR) and Ln(proteinuria).

**Table 3:** Multivariate logistic regression of kidney injury status as assessed by eGFR, proteinuria and the KIT Score.

Parameter	Estimate	df	s.e.	χ^2^	*p*-Value	OR	2.5%	97.5%
Intercept	−27.1034	1	29.8369	0.8245	0.3637			
eGFR	3.5354	1	5.5596	0.4045	0.5248	34.3	2.91 × 10^−4^	8.38 × 10^6^
Proteinuria	1.3151	1	0.8043	2.6732	0.1020	3.73	1.01	27.22
KIT Score	0.7847	1	0.2834	7.6674	0.0056	2.19	1.48	4.75

Model likelihood ratio $\chi_{3}^{2}$ = 92.5844, *p*-value < 0.0001. OR is odds ratio for increased of kidney injury per unit change in Ln(eGFR), Ln(proteinuria) and unit change in KIT Scored (scaling from 0 to 100).

**Table 4:** Multivariate logistic regression of kidney injury status as assessed by individual KIT urine biomarkers.

Parameter	Estimate	df	s.e.	χ^2^	*p*-Value	OR
Intercept	56.0716	1	12.9866	18.6451	<0.0001	
eGFR	−12.5302	1	2.7181	21.2521	<0.0001	3.62 × 10^−6^
Urine cfDNA	−0.2720	1	0.0973	7.8120	0.0052	0.76
Urine m-cfDNA	−1.1260	1	0.2655	17.9946	<0.0001	0.32
Urine protein	0.7976	1	0.1815	19.3702	<0.0001	2.22
Urine CXCL10	1.1304	1	0.5216	4.6959	0.0302	3.10
Urine clusterin	−0.3506	1	0.2538	1.9099	0.1671	0.70
Urine creatinine	0.6448	1	0.5613	1.3202	0.2506	1.91

OR is odds ratio for increased risk of kidney injury per unit change on Ln scale.

**Table 5:** Distribution of mean KIT Scores and presence/absence of proteinuria by CKD stage.

CKD Stage by eGFR	Mean KIT Score/CKD Stage	% Patients w/o Proteinuria	% Patients w/o Proteinuria Who Have High-Risk for Kidney Injury (KIT Score > 18.5)
CKD Stage 1	24.4	66% (84/127)	91% (77/84)
CKD Stage 2	35.3	60% (53/87)	100%
CKD Stage 3	48.4	47% (38/80)	100%
CKD Stage 4	66.9	8% (3/36)	100%
CKD Stage 5	90.8	7% (1/13)	100%

Proteinuria becomes more predictive for kidney injury with advancing CKD stages and the progression of kidney injury. Recognized as a late marker of kidney injury, proteinuria becomes more invariant in CKD 4–5. In earlier stages of CKD, the KIT Score detects kidney injury, independent of proteinuria.
